# Supplementary material for: Comprehensive Annotation and Functional Exploration of MicroRNAs in Lettuce
Source: Front Plant Sci. 2021 Dec 24;12:781836. doi: 10.3389/fpls.2021.781836 (PMC8739914; doi:10.3389/fpls.2021.781836)
Supplement: Supplementary file 2 [file Data_Sheet_8.ZIP › GSM2230749.plot/Lsa-miR408_Lsat_1_v5_gn_9_73760.1_660_TPlot.pdf]

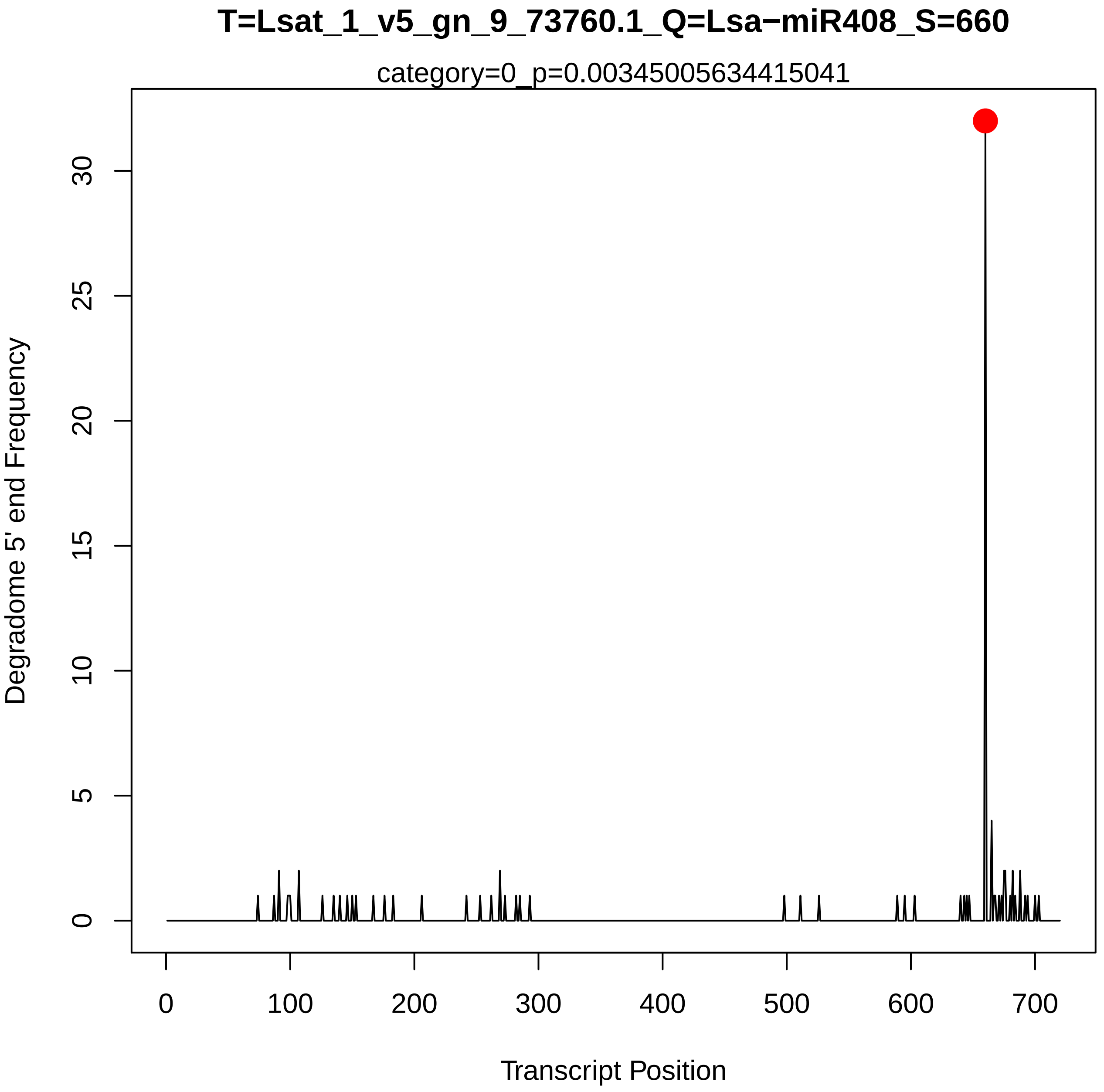
T=Lsat_1_v5_gn_9_73760.1_Q=Lsa−miR408_S=660
category=0_p=0.00345005634415041
Degradome 5' end Frequency
0
5
10
15
20
25
30
0
100
200
300
400
500
600
700
Transcript Position
